# Supplementary material for: Circulating Tumour DNA Guided Adjuvant Chemotherapy Decision Making in Stage II Colon Cancer—A Clinical Vignette Study
Source: Cancers (Basel). 2023 Oct 31;15(21):5227. doi: 10.3390/cancers15215227 (PMC10648421; doi:10.3390/cancers15215227)
Supplement: Supplementary file 1 [file cancers-15-05227-s001.zip › cancers-2662624-supplementary.pdf]

1. Respondents' demographics

Table S1 demonstrates the respondents' demographics. For later analyses, medical oncologists with ≤10 years experiences were considered early career and medical oncologists who see ≤ 10 new stage II colon cancer (CC) patients per year were considered to see a limited number of new patients.

Table S1: Demographics

	Overall (n = 64)
Country, *n (%)*	
• Australia	45 (70.3%)
• Canada	13 (20.3%)
• New Zealand	6 (9.4%)
Primary role held in, *n (%)*	
• Rural/regional practice	18 (28.1%)
• Private practice	13 (20.3%)
• Specialised oncologist centre	19 (29.7 %)
Primarily practices as clinician, *n (%)*	62 (96.9%)
Years as qualified medical oncologist, *n (%)*	
• <5 years	9 (14.1%)
• 5 to 10 years	14 (21.9%)
• 11 to 20 years	28 (43.8%)
• >20 years	13 (20.3%)
Number of new stage II colon cancer patients treated per year, *n (%)*	
• 0 to 5	18 (28.1%)
• 6 to 10	21 (32.8%)
• 11 to 15	12 (18.8%)
• > 15	13 (20.3%)

2. Pre-ctDNA adjuvant recommendations stratified by age and risk

A series of paired Chi-square tests was conducted to compare the proportion of oncologist recommending adjuvant chemotherapy (AC) stratified by clinicopathological risk level and age (Table S2). Results demonstrate that proportions significantly changed when considering clinicopathological risk. When considering age, the proportion significantly changed when comparing elderly patients (≥ 70 years) to other age groups. When considering only high risk patients, there was a significant difference between elderly patients (≥ 70 years) and younger patients (<50 years).

Table S2: Results of paired Chi-square testing comparing proportion of oncologists recommending AC.

Comparison	p-value
Stratified by risk	
• Low vs. Intermediate	<0.01
• Low vs. High	<0.01
• Intermediate vs. High	<0.01

Stratified by age	
• ≤ 50 vs. 51 to 69 years	0.23
• ≤ 50 vs. ≥ 70 years	<0.01
• 51 to 60 vs. ≥ 70 years	0.08
Stratified by age (intermediate risk vignettes only)	
• ≤ 50 vs. 51 to 69 years	0.15
• ≤ 50 vs. ≥ 70 years	<0.01
• 51 to 60 vs. ≥ 70 years	<0.01
Stratified by age (high risk vignettes only)	
• ≤ 50 vs. 51 to 69 years	0.13
• ≤ 50 vs. ≥ 70 years	0.02
• 51 to 60 vs. ≥ 70 years	0.58

3. Demographics as predictor of AC recommendation (pre-ctDNA testing)

Pre-ctDNA testing, there was no difference in the proportion of medical oncologists recommending AC in all vignettes when stratifying career, new patients seen per year, and type of practice. (Table S3).

Table S3: Comparison of oncologists recommending adjuvant chemotherapy stratified by demographic factors (pre-ctDNA testing)

Clinico-pathological risk	Age	Early (<10 years) career, *n (%)*	Later (≥ 10 years) career, *n (%)*	p-value
Low	≤ 50	1 (4%)	2 (5%)	0.82
	51 to 69	0 (0%)	0 (0%)	NA
	≥ 70	0 (0%)	0 (0%)	NA
Intermediate	≤ 50	12 (63%)	30 (75%)	0.35
	51 to 69	12 (52%)	24 (60%)	0.55
	≥ 70	6 (30%)	13 (33%)	0.84
High	≤ 50	20 (100%)	60 (100%)	NA
	51 to 69	19 (95%)	37 (93%)	0.71
	≥ 70	20 (87%)	36 (90%)	0.71
		Limited (<10) new patients/year, *n (%)*	Many (≥ 10) new patients/year, *n (%)*	
Low	≤ 50	2 (5%)	1 (4%)	0.84
	51 to 69	0 (0%)	0 (0%)	NA
	≥ 70	0 (0%)	0 (0%)	NA
Intermediate	≤ 50	24 (69%)	18 (75%)	0.69
	51 to 69	21 (54%)	15 (63%)	0.50
	≥ 70	9 (25%)	10 (42%)	0.17
High	≤ 50	36 (100%)	24 (100%)	NA
	51 to 69	32 (89%)	24 (100%)	0.09
	≥ 70	35 (90%)	21 (88%)	0.78

		Private practice, *n (%)*	Public practice, *n (%)*	
Low	≤ 50	1 (8%)	2 (4%)	0.57
	51 to 69	0 (0%)	0 (0%)	NA
	≥ 70	0 (0%)	0 (0%)	NA
Intermediate	≤ 50	8 (67%)	34 (72%)	0.70
	51 to 69	6 (50%)	30 (59%)	0.58
	≥ 70	4 (33%)	15 (31%)	0.89
High	≤ 50	12 (100%)	48 (100%)	NA
	51 to 69	10 (83%)	46 (96%)	0.12
	≥ 70	10 (83%)	46 (90%)	0.50
		Rural/regional practice, *n (%)*	Metropolitan practice, *n (%)*	
Low	≤ 50	1 (6%)	2 (4%)	0.84
	51 to 69	0 (0%)	0 (0%)	NA
	≥ 70	0 (0%)	0 (0%)	NA
Intermediate	≤ 50	11 (69%)	31 (72%)	0.80
	51 to 69	9 (50%)	27 (60%)	0.47
	≥ 70	4 (25%)	15 (34%)	0.50
High	≤ 50	16 (100%)	44 (100%)	NA
	51 to 69	15 (94%)	41 (96%)	0.94
	≥ 70	17 (94%)	39 (87%)	0.38

4. Demographics as predictor of AC recommendation (ctDNA negative)

Following a ctDNA negative result, there was no difference in the proportion of medical oncologists recommending AC in all vignettes when stratifying career, new patients seen per year, and type of practice. (Table S4).

Table S4: Comparison of oncologists recommending adjuvant chemotherapy stratified by demographic factors (ctDNA negative)

Clinico-pathological risk	Age	Early (<10 years) career, *n (%)*	Later (≥ 10 years) career, *n (%)*	p-value
Low	≤ 50	0 (0%)	0 (0%)	NA
	51 to 69	0 (0%)	0 (0%)	NA
	≥ 70	0 (0%)	0 (0%)	NA
Intermediate	≤ 50	3 (16%)	3 (8%)	0.33
	51 to 69	0 (0%)	0 (0%)	NA
	≥ 70	0 (0%)	0 (0%)	NA
High	≤ 50	11 (55%)	19 (48%)	0.58
	51 to 69	8 (40%)	14 (35%)	0.71
	≥ 70	5 (23%)	11 (28%)	0.68
		Limited (<10) new patients/year, *n (%)*	Many (≥ 10) new patients/year, *n (%)*	
Low	≤ 50	0 (0%)	0 (0%)	NA

	51 to 69	0 (0%)	0 (0%)	NA
	≥ 70	0 (0%)	0 (0%)	NA
Intermediate	≤ 50	4 (11%)	2 (8%)	0.70
	51 to 69	0 (0%)	0 (0%)	NA
	≥ 70	0 (0%)	0 (0%)	NA
High	≤ 50	18 (50%)	12 (50%)	1.00
	51 to 69	13 (36%)	9 (38%)	0.91
	≥ 70	8 (21%)	8 (33%)	0.28
		Private practice, *n (%)*	Public practice, *n (%)*	
Low	≤ 50	0 (0%)	0 (0%)	NA
	51 to 69	0 (0%)	0 (0%)	NA
	≥ 70	0 (0%)	0 (0%)	NA
Intermediate	≤ 50	1 (8%)	5 (11%)	0.81
	51 to 69	0 (0%)	0 (0%)	NA
	≥ 70	0 (0%)	0 (0%)	NA
High	≤ 50	4 (33%)	26 (54%)	0.20
	51 to 69	2 (17%)	20 (42%)	0.11
	≥ 70	1 (8%)	15 (30%)	0.12
		Rural/regional practice, *n (%)*	Metropolitan practice, *n (%)*	
Low	≤ 50	0 (0%)	0 (0%)	NA
	51 to 69	0 (0%)	0 (0%)	NA
	≥ 70	0 (0%)	0 (0%)	NA
Intermediate	≤ 50	3 (19%)	3 (7%)	0.18
	51 to 69	0 (0%)	0 (0%)	NA
	≥ 70	0 (0%)	0 (0%)	NA
High	≤ 50	8 (50%)	22 (50%)	1.00
	51 to 69	7 (44%)	15 (34%)	0.49
	≥ 70	6 (33%)	10 (23%)	0.39

5. Demographics as predictor of AC recommendation (ctDNA positive)

Following, a positive ctDNA result, there was no difference in the proportion of medical oncologists recommending AC in all vignettes when stratifying career, new patients seen per year, and type of practice. (Table S5).

Table S5: Comparison of oncologists recommending adjuvant chemotherapy stratified by demographic factors (ctDNA positive)

Clinico-pathological risk	Age	Early (<10 years) career, *n (%)*	Later (≥ 10 years) career, *n (%)*	p-value
Low	≤ 50	20 (87%)	39 (95%)	0.23
	51 to 69	16 (84%)	38 (95%)	0.16
	≥ 70	17 (85%)	38 (95%)	0.19
Intermediate	≤ 50	17 (90%)	38 (95%)	0.43

	51 to 69	21 (91%)	38 (95%)	0.56
	≥ 70	18 (90%)	38 (95%)	0.46
High	≤ 50	20 (100%)	40 (100%)	NA
	51 to 69	20 (100%	40 (100%)	NA
	≥ 70	22 (100%	40 (100%)	NA
		Limited (<10) new patients/year, *n (%)*	Many (≥ 10) new patients/year, *n (%)*	
Low	≤ 50	36 (92%)	23 (92%)	0.96
	51 to 69	32 (91%)	22 (92%)	0.97
	≥ 70	33 (92%)	22 (92%)	1.00
Intermediate	≤ 50	33 (94%)	22 (92%)	0.69
	51 to 69	37 (95%)	22 (92%)	0.61
	≥ 70	34 (94%)	22 (92%)	0.67
High	≤ 50	36 (100%)	24 (100%)	NA
	51 to 69	36 (100%)	24 (100%)	NA
	≥ 70	38 (100%)	24 (100%)	NA
		Private practice, *n (%)*	Public practice, *n (%)*	
Low	≤ 50	13 (100%)	46 (90.2%)	0.24
	51 to 69	12 (100%)	42 (89%)	0.24
	≥ 70	11 (92%)	44 (92%)	1.00
Intermediate	≤ 50	12 (100%)	47 (92%)	0.30
	51 to 69	12 (100%)	47 (92%)	0.33
	≥ 70	12 (100%)	44 (91.7%)	0.30
High	≤ 50	12 (100%)	48 (100%)	NA
	51 to 69	12 (100%)	48 (100%)	NA
	≥ 70	12 (100%)	50 (100%)	NA
		Rural/regional practice, *n (%)*	Metropolitan practice, *n (%)*	
Low	≤ 50	16 (89%)	43 (94%)	0.54
	51 to 69	13 (81%)	41 (95%)	0.08
	≥ 70	13 (81%)	42 (96%)	0.08
Intermediate	≤ 50	14 (88%)	41 (95%)	0.29
	51 to 69	16 (89%)	43 (96%)	0.33
	≥ 70	14 (88%)	42 (96%)	0.28
High	≤ 50	16 (100%)	44 (100%)	NA
	51 to 69	16 (100%)	44 (100%)	NA
	≥ 70	18 (100%)	44 (100%)	NA

6. Adjuvant chemotherapy recommendations following ctDNA testing

Table S6 demonstrates the specific AC regimens recommended by oncologists based on ctDNA results.

Table S6: Adjuvant chemotherapy regimen recommendations following ctDNA testing.

Vignette	Low risk						Intermediate risk						High risk					
	≤ 50		51 to 69		≥ 70		≤ 50		51 to 69		≥ 70		≤ 50		51 to 69		≥ 70	
ctDNA	**Neg**	**Pos**	**Neg**	**Pos**	**Neg**	**Pos**	**Neg**	**Pos**	**Neg**	**Pos**	**Neg**	**Pos**	**Neg**	**Pos**	**Neg**	**Pos**	**Neg**	**Pos**
No.	64	64	59	59	60	60	59	59	63	63	60	60	60	60	60	60	62	62
Recommend AC	0% (0)	92.2% (59)	0% (0)	91.5% (54)	0% (0)	91.7% (55)	10.2% (6)	93.2% (55)	0% (0)	93.7% (59)	0% (0)	93.3% (56)	50% (30)	100% (60)	36.7% (22)	100% (60)	25.8% (16)	100% (62)
Regimen																		
3M Single	0% (0)	4.7% (3)	0% (0)	1.7% (1)	0% (0)	5% (3)	3.4% (2)	0% (0)	0% (0)	0% (0)	0% (0)	1.7% (1)	1.7% (1)	0% (0)	1.7% (1)	0% (0)	1.6% (1)	1.6% (1)
6M Single	0% (0)	29.7% (19)	0% (0)	28.8% (17)	0% (0)	58.3% (35)	3.4% (2)	23.7% (14)	0% (0)	22.2% (14)	0% (0)	51.7% (31)	26.7% (16)	18.3% (11)	16.7% (10)	23.3% (14)	17.7% (11)	48.4% (30)
3M Doublet	0% (0)	28.2% (18)	0% (0)	32.2% (19)	0% (0)	10% (6)	3.4% (2)	35.6% (21)	0% (0)	34.9% (22)	0% (0)	18.3% (11)	18.3% (11)	33.3% (20)	15% (9)	31.7% (19)	4.8% (3)	17.7% (11)
6M Doublet	0% (0)	29.7% (19)	0% (0)	28.8% (17)	0% (0)	18.3% (11)	0% (0)	33.9% (20)	0% (0)	36.5% (23)	0% (0)	21.7% (13)	3.3% (2)	48.3% (29)	3.3% (2)	45% (27)	1.6% (1)	32.3% (20)
No Chemo	100% (64)	7.8% (5)	100% (59)	8.5% (5)	100% (60)	8.3% (5)	89.8% (53)	6.8% (4)	100% (63)	6.4% (4)	100% (60)	6.7% (4)	50% (30)	0% (0)	63.3% (38)	0% (0)	74.2% (46)	0% (0)

7. De-escalation and escalation of treatment

Table S7 and S8 demonstrates the number of oncologists that de-escalated or escalated their AC recommendation following a negative and positive ctDNA result. The following definitions were utilised:

- Escalation: Oncologists were considered to have escalated treatment if following ctDNA testing, any of the following recommendations were made:
  - Change from no AC to AC
  - Change from single agent AC to doublet AC
  - Prolonged duration from 3 to 6 months
- De-escalation: Oncologists were considered to have de-escalated treatment if following ctDNA testing, any of the following recommendations were made:
  - Change from AC to no AC
  - Change from doublet AC to single agent AC
  - Shortened duration from 6 to 3 months

If an oncologist recommended changing from single to doublet but shortened duration (i.e. escalating agent but de-escalating duration), this was considered an escalation. Conversely, if they changed from doublet to single but increased duration (i.e. de-escalating agent but escalating duration), this was considered a de-escalation.

Table S7: Number of oncologists recommending changes in AC following a negative ctDNA result.

Vignette	**Low risk**			**Intermediate risk**			**High risk**		
	**≤ 50**	**51 to 69**	**≥ 70**	**≤ 50**	**51 to 69**	**≥ 70**	**≤ 50**	**51 to 69**	**≥ 70**
No change	61	59	60	21	27	41	22	20	22
De-escalate	3	0	0	38	36	19	38	40	41
Escalate	0	0	0	0	0	0	0	0	0

Table S8: Number of oncologists recommending changes in AC following a positive ctDNA result.

Vignette	**Low risk**			**Intermediate risk**			**High risk**		
	**≤ 50**	**51 to 69**	**≥ 70**	**≤ 50**	**51 to 69**	**≥ 70**	**≤ 50**	**51 to 69**	**≥ 70**
No change	5	5	5	22	15	13	37	33	38
De-escalate	0	0	0	0	0	0	0	0	0
Escalate	59	54	55	37	48	47	23	27	25

8. Checklist for reporting results of Internet E-surveys

The responses to the Checklist for Reporting Results of Internet E-surveys (CHERRIES) is provided below in Table S9 to ensure consistency and completeness of reporting.[1]

Table S9: Responses to the Checklist for Reporting Results of Internet E-surveys (CHERRIES)

**Item category**	**Checklist item**	**Explanation**
Design	Describe survey design	A clinical vignette survey designed to elicit responses from medical oncologists who treat colon cancers. Medical oncologists were presented with vignettes composed of two variables with three levels each. This resulted in each respondent being asked to complete 9 vignettes in total. Basic demographic and attitudes/understanding of ctDNA testing were also collected.<br><br>A convenience example of medical oncologists from Australian, Canada, and New Zealand were approached as each countries' model of health care is similar, specifically being public taxpayer funded health care systems.
International review board (IRB) approval and informed consent process	IRB approval	The survey was granted approval from the Human Research Ethics Committee based at Walter and Eliza Hall Institute of Medical Research (Melbourne, Australia).
	Informed consent	Informed consent was embedded into the survey. Respondents were presented with an explanation of the purpose of the study, the involved investigators, and the estimated time lengths at the beginning of the survey. Respondents had to provide consent before proceeding with the survey.<br><br>Additional information was also provided in the electronic mail that was used to approach potential participants.
	Data collection	The survey was developed in the Qualtrics Research Core platform hosted by the University of Melbourne, Australia. All data was hosted and stored on this platform and password protected with access only allowed to the corresponding author.<br><br>No personal demographic information was collected from participants such as name or date of birth. Only broad demographics related to their professional experience, e.g. the length of time they have been qualified as a medical oncologist, were collected.
Development and pre-testing	Development and testing	The survey was developed on the Qualtrics Research Core platform and extensively tested by the corresponding and co-authors for

		functionality prior to fielding of the survey to participants. Useability on computers and mobile devices were tested as part of this process.<br><br>A small pilot was conducted prior to fielding with three medical oncologist invited to trial the survey prior to fielding to potential participants.
Recruitment process and description of the sample having access to the questionnaire	Open survey vs. closed survey	This study was an open study.
	Contact method	Respondents were identified through membership to various professional bodies: the Medical Oncology Group of Australia (MOGA), Canadian Cancer Trials Group (CCTG), and New Zealand Society for Oncology (NZSO). The surview were reviewed by each committee prior to fielding.<br><br>Electronic mailing lists were provided by each professional body and participants were contacted by an introductory electronic mail with an embedded hyperlink to the survey.
	Advertising	No advertising campaign was undertaken. Potential participants were made aware of the survey by the way of the introductory electronic mail.
Survey administration	Web/e-mail	Participants completed the survey on the cloud-based Qualtrics platform with the data being stored on the platform.
	Context	The survey was hosted on the Qualtrics platform.
	Mandatory/voluntary	Participant was purely voluntary and there were no mechanisms to enforce participation within the survey platform.
	Incentives	No financial incentives were offered.
	Time/Date	Australian participants were invited in November 2022. Canadian and New Zealander participants were invited in February and March 2023, respectively. The survey was closed in June 2023.
	Randomisation of items or questionnaires	Participants were presented with all demographic questions and clinical vignette scenarios.<br><br>It was noted after initial fielding of the study to Australian participants that the last 2 – 3 clinical vignettes had a lower rate of response due to incomplete surveys. The order of the clinical vignettes were reversed when fielded to Canadian and New Zealander participants to

		offset this. However, participants were presented with all vignettes.
	Adaptive questioning	The clinical vignettes had adaptive questioning. If participants elected to not recommend adjuvant chemotherapy then no further questions were asked regarding specific regimen or duration. Conversely, if adjuvant chemotherapy were recommended then questions regarding specific regimen or duration would be presented.
	Number of items	The demographic data fields consisted of 4 items. The understanding and attitudes to ctDNA testing consisted of 5 items.<br><br>The clinical vignettes consisted of 9 clinical scenarios, with a maximum potential of 10 items for each vignette. As previously noted, this was adaptive so depending on responses not all 10 items were presented. Despite the length of clinical vignettes, the structure of questions were similar and in the pilot testing step, it was felt to be appropriate and not difficult to complete.
	Number of screens	In total, 15 screens were presented.
	Completeness check	The study was designed that the participant could not proceed to the next screen until all questions were responded to. Selection of one answer only was enforced in the clinical vignettes.
	Review step	Participants were allowed to return to previous screens to edit responses prior to submission.
Response rates	Unique site visitor	Unique site visitor data was not collected.
	View rate	The survey was hosted on the Qualtrics Research Core research and only accessible through the hyperlink embedded in the introductory e-mail. The platform does not have a tool to allow calculation of the number of participants that accessed the first screen of the survey so a rate cannot be calculated.
	Participation rate	Estimated 8.8% based on total number of oncologists the introductory e-mail was circulated to (n = 64 responses / 721 medical oncologists). However, for the Australian cohort (n = 396), the survey were circulated to all medical oncologists with membership to MOGA and not specifically to oncologists who treat colon cancers to the total number that could have answered the survey is difficult to estimate. Additionally, the mailing list for New Zealand included surgeons as well as medical oncologist asking make the total number of potential respondents difficult ot estimate.

		A breakdown of response rates by country is as follows: Australia (13%; 45/396), Canada (5%; 13/242), and New Zealand (7.2%; 7/83).
	Completion rate	92% of respondents completed all 9 vignettes.
Preventing multiple entries from same individual	Cookies used	The Qualtrics Research Core platform assigns specific identification number based on IP address. Cookies are used to retrieve incomplete surveys for participants to continue responding to. Specific details of these cookies are available here: https://www.qualtrics.com/cookie-statement/
	IP check	The Qualtrics Research Core platform assigns specific identification number based on IP address. IP checks were completed prior to data analysis to ensure there were no duplicates. No duplicates were detected but if duplicates had been detected, the protocol would have been to select the most completed response to analyse.
	Log file analysis	No other methods were used beyond checking of IP addresses.
	Registration	This survey was an open design so no registration was required.
Analysis	Handling of incomplete responses	Each clinical vignette were analysed independently. If a vignette had incomplete responses, the response for that vignette was discarded.
	Questions submitted with an atypical timestamp	The Qualtrics Research Core platform records the time taken to complete the survey in seconds. The average time of completion was 724 second (approximately 12 minutes). Responses that were completed in under 300 seconds (5 minutes) were manually reviewed to ensure appropriate completion of items. This was based on completion time by the corresponding and co-authors who had greatest familiarity with the survey.
	Statistical correction	Weighting and propensity scoring was not carried out in the analysis.

9. **Full survey**

The full survey is presented below:

9.1 Preface

You are invited to participate in an online research study about the role that circulating tumour DNA (ctDNA) may play in guiding adjuvant chemotherapy decision making in stage II colon cancer. The goal of this survey is to determine how oncologists will utilise ctDNA in clinical practice through a series of nine patient vignettes.

The survey is designed to be completed by qualified medical oncologists who are familiar with the treatment of stage II colon cancer patients. It is anticipated that the survey will take no longer than 15 minutes to complete.

This study is being conducted by Dr. Yat Hang To under the supervision of Associate Professor Jeanne Tie, Dr. Koen Degeling and Professor Peter Gibbs. The results will inform subsequent health economic modelling that will examine the cost-effectiveness of ctDNA in an Australian context. Demonstration of cost-effectiveness may assist in later submission to Australian regulatory bodies to secure reimbursement and enable widespread access to ctDNA testing.

Participation in this study is voluntary. No identifying information such as name or date of birth will be collected so participants and responses will remain anonymous. Study findings will be presented only in summary form and no identifying information will be used in any report.

If you have any questions about this study, please contact Dr. Yat Hang To (REDACTED).

Please indicate if you consent to participate in this study

- Yes
- No

9.2 Introduction

Circulating tumour DNA (ctDNA) is a blood-based biomarker that has been demonstrated to be an independent and prognostic marker of recurrence compared to traditional clinicopathological parameters in stage II colorectal cancer (CRC).

Recently, the DYNAMIC study investigated its use in stage II CRC, randomising patients to either a ctDNA guided approach or standard of care approach to adjuvant chemotherapy decision making. Patients had their ctDNA status determined post-surgery with those randomised to the biomarker-driven arm being prescribed chemotherapy based on the results.

The results of DYNAMIC study can be accessed here: https://www.nejm.org/doi/full/10.1056/NEJMoa2200075

9.3 Part 1: Demographics

1. Please indicate the country in which you primarily practice:
    a. Australia
    b. New Zealand
    c. Canada
2. Please select the terms that best describes where your primary place of practice
    a. Metropolitan
    b. Regional/Rural
    c. Private
    d. Public
    e. General hospital
    f. Oncology centre
3. Please indicate the number of years you have been a qualified oncologist:
    a. <5 years
    b. 5 to 10 years
    c. 10 to 20 years
    d. >20 years
4. Please estimate the number of patients with a new diagnosis of stage II colon cancer you treat per year:
    a. 0 to 5
    b. 6 to 10
    c. 11 to 15
    d. >15

9.4 Part 2: Concept of ctDNA

1) Are you familiar with the concept of circulating tumour DNA?
    a. Yes
    b. No
2) What is your view on the statement "Patients with detectable ctDNA following resection of stage II colon cancer are at higher risk of recurrence compared to patients with undetectable ctDNA":
    a. Strongly agree
    b. Agree
    c. Unsure
    d. Disagree
    e. Strongly disagree
3) Prior to this survey, I was aware of the publication of the DYNAMIC study:
    a. Yes
    b. No
4) I have read the DYNAMIC study publication:
    a. Yes
    b. No
5) If ctDNA testing was commercially available but not reimbursed by taxpayer funds, what is the maximum cost that you believe a patient should pay for ctDNA testing (i.e. patients paying privately for testing)?
    a. Sliding scale ranging from $0 to $10,000

9.5 Part 3: Clinical vignettes

Introduction to clinical vignettes

You will now be presented with a series of clinical vignettes describing patients with newly diagnosed stage II colon cancer who are being assessed for their suitability for adjuvant chemotherapy. You will be asked a series questions regarding your management of these patients before and following ctDNA testing.

All patients present with Eastern Co-operative Oncology Group Performance Status (ECOG) of 0 to 1.

The stratification system for presenting high-risk clinicopathological features for recurrence have been adapted from the ESMO Clinical Practice Guidelines for Localised Colon Cancer:

**High risk**	Presents with **ANY** of the following features:<br>1. <12 lymph nodes examined<br>2. pT4 stage including perforation<br>3. Multiple (>1) intermediate risk features
**Intermediate risk**	Presents with **ONLY ONE** of the following features:<br>1. Poor/high grade tumour differentiation<br>2. Lymphovascular invasion<br>3. Perineural invasion<br>4. Tumour obstruction<br>**OR**<br>1. Mismatch repair proficient (pMMR) with only ONE of the above features
**Low risk**	Presents with:<br>1. pMMR or MMR deficient (dMMR) with NO high risk or intermediate features<br>**OR**<br>2. dMMR with only ONE intermediate risk feature

Vignette 1:
Patient A is ≤ **50 years old** with a recently resected stage II colon cancer. Clinicopathological risk for recurrence: **Low risk**

1) Would you prescribe adjuvant chemotherapy to Patient A?
    a. Yes – *please note question 2 and 3 only becomes visible if "Yes" is selected*
    b. No
2) Please select the chemotherapy regimen you would recommend for Patient A:
    a. 5-FU
    b. Capecitabine
    c. FOLFOX
    d. CAPOX
3) Please select the duration of chemotherapy you would recommend:
    a. 3 months
    b. 6 months
4) Following surgery, for Patient A would you order ctDNA testing if publicly available?
    a. Yes

    b. No
5) Patient A's ctDNA status was tested following surgery. If the patient's ctDNA status was **negative** would you prescribe adjuvant chemotherapy?
    a. Yes
    b. No
6) Please select the chemotherapy regimen you would recommend to Patient A if the ctDNA status was **negative**:
    a. 5-FU
    b. Capecitabine
    c. FOLFOX
    d. CAPOX
7) Please select the duration of chemotherapy you would recommend:
    a. 3 months
    b. 6months
8) Patient A's ctDNA status was tested following surgery. If the patient's ctDNA status was **positive** would you prescribe adjuvant chemotherapy?
    a. Yes
    b. No
9) Please select the chemotherapy regimen you would recommend to Patient A if the ctDNA status was **positive**:
    a. 5-FU
    b. Capecitabine
    c. FOLFOX
    d. CAPOX
10) Please select the duration of chemotherapy you would recommend:
    a. 3 months
    b. 6months

Vignette 2:

Patient B is **between 51 to 69 years old** with a recently resected stage II colon cancer. Clinicopathological risk for recurrence: **Intermediate risk**

1) Would you prescribe adjuvant chemotherapy to Patient B?
    a. Yes – *please note question 2 and 3 only becomes visible if "Yes" is selected*
    b. No
2) Please select the chemotherapy regimen you would recommend for Patient B:
    a. 5-FU
    b. Capecitabine
    c. FOLFOX
    d. CAPOX
3) Please select the duration of chemotherapy you would recommend:
    a. 3 months
    b. 6 months
4) Following surgery, for Patient B would you order ctDNA testing if publicly available?
    a. Yes
    b. No
5) Patient B's ctDNA status was tested following surgery. If the patient's ctDNA status was **negative** would you prescribe adjuvant chemotherapy?
    a. Yes
    b. No

6) Please select the chemotherapy regimen you would recommend to Patient B if the ctDNA status was **negative**:
    a. 5-FU
    b. Capecitabine
    c. FOLFOX
    d. CAPOX
7) Please select the duration of chemotherapy you would recommend:
    a. 3 months
    b. 6months
8) Patient B's ctDNA status was tested following surgery. If the patient's ctDNA status was **positive** would you prescribe adjuvant chemotherapy?
    a. Yes
    b. No
9) Please select the chemotherapy regimen you would recommend to Patient B if the ctDNA status was **positive**:
    a. 5-FU
    b. Capecitabine
    c. FOLFOX
    d. CAPOX
10) Please select the duration of chemotherapy you would recommend:
    a. 3 months
    b. 6months

Vignette 3:
Patient C is $\geq$ **70 years old** with a recently resected stage II colon cancer. Clinicopathological risk for recurrence: **High risk**

1) Would you prescribe adjuvant chemotherapy to Patient C?
    a. Yes – *please note question 2 and 3 only becomes visible if "Yes" is selected*
    b. No
2) Please select the chemotherapy regimen you would recommend for Patient C:
    a. 5-FU
    b. Capecitabine
    c. FOLFOX
    d. CAPOX
3) Please select the duration of chemotherapy you would recommend:
    a. 3 months
    b. 6 months
4) Following surgery, for Patient C would you order ctDNA testing if publicly available?
    a. Yes
    b. No
5) Patient C's ctDNA status was tested following surgery. If the patient's ctDNA status was **negative** would you prescribe adjuvant chemotherapy?
    a. Yes
    b. No
6) Please select the chemotherapy regimen you would recommend to Patient C if the ctDNA status was **negative**:
    a. 5-FU
    b. Capecitabine
    c. FOLFOX
    d. CAPOX

7) Please select the duration of chemotherapy you would recommend:
    a. 3 months
    b. 6months
8) Patient C's ctDNA status was tested following surgery. If the patient's ctDNA status was **positive** would you prescribe adjuvant chemotherapy?
    a. Yes
    b. No
9) Please select the chemotherapy regimen you would recommend to Patient C if the ctDNA status was **positive**:
    a. 5-FU
    b. Capecitabine
    c. FOLFOX
    d. CAPOX
10) Please select the duration of chemotherapy you would recommend:
    a. 3 months
    b. 6months

Vignette 4:
Patient D is **<u><</u> 50 years old** with a recently resected stage II colon cancer. Clinicopathological risk for recurrence: **High risk**

1) Would you prescribe adjuvant chemotherapy to Patient D?
    a. Yes – *please note question 2 and 3 only becomes visible if "Yes" is selected*
    b. No
2) Please select the chemotherapy regimen you would recommend for Patient D:
    a. 5-FU
    b. Capecitabine
    c. FOLFOX
    d. CAPOX
3) Please select the duration of chemotherapy you would recommend:
    a. 3 months
    b. 6 months
4) Following surgery, for Patient D would you order ctDNA testing if publicly available?
    a. Yes
    b. No
5) Patient D's ctDNA status was tested following surgery. If the patient's ctDNA status was **negative** would you prescribe adjuvant chemotherapy?
    a. Yes
    b. No
6) Please select the chemotherapy regimen you would recommend to Patient D if the ctDNA status was **negative**:
    a. 5-FU
    b. Capecitabine
    c. FOLFOX
    d. CAPOX
7) Please select the duration of chemotherapy you would recommend:
    a. 3 months
    b. 6months
8) Patient D's ctDNA status was tested following surgery. If the patient's ctDNA status was **positive** would you prescribe adjuvant chemotherapy?

a. Yes
b. No

9) Please select the chemotherapy regimen you would recommend to Patient D if the ctDNA status was **positive**:
    a. 5-FU
    b. Capecitabine
    c. FOLFOX
    d. CAPOX
10) Please select the duration of chemotherapy you would recommend:
    a. 3 months
    b. 6months

Vignette 5:
Patient E is ≥ **70 years old** with a recently resected stage II colon cancer. Clinicopathological risk for recurrence: **Low risk**

1) Would you prescribe adjuvant chemotherapy to Patient E?
    a. Yes – *please note question 2 and 3 only becomes visible if "Yes" is selected*
    b. No
2) Please select the chemotherapy regimen you would recommend for Patient E:
    a. 5-FU
    b. Capecitabine
    c. FOLFOX
    d. CAPOX
3) Please select the duration of chemotherapy you would recommend:
    a. 3 months
    b. 6 months
4) Following surgery, for Patient E would you order ctDNA testing if publicly available?
    a. Yes
    b. No
5) Patient E's ctDNA status was tested following surgery. If the patient's ctDNA status was **negative** would you prescribe adjuvant chemotherapy?
    a. Yes
    b. No
6) Please select the chemotherapy regimen you would recommend to Patient E if the ctDNA status was **negative**:
    a. 5-FU
    b. Capecitabine
    c. FOLFOX
    d. CAPOX
7) Please select the duration of chemotherapy you would recommend:
    a. 3 months
    b. 6months
8) Patient E's ctDNA status was tested following surgery. If the patient's ctDNA status was **positive** would you prescribe adjuvant chemotherapy?
    a. Yes
    b. No
9) Please select the chemotherapy regimen you would recommend to Patient E if the ctDNA status was **positive**:
    a. 5-FU
    b. Capecitabine

c. FOLFOX
   d. CAPOX
10) Please select the duration of chemotherapy you would recommend:
   a. 3 months
   b. 6months

Vignette 6:
Patient F is **between 51 to 69 years old** with a recently resected stage II colon cancer.
Clinicopathological risk for recurrence: **High risk**

1) Would you prescribe adjuvant chemotherapy to Patient F?
   a. Yes – *please note question 2 and 3 only becomes visible if "Yes" is selected*
   b. No
2) Please select the chemotherapy regimen you would recommend for Patient F:
   a. 5-FU
   b. Capecitabine
   c. FOLFOX
   d. CAPOX
3) Please select the duration of chemotherapy you would recommend:
   a. 3 months
   b. 6 months
4) Following surgery, for Patient F would you order ctDNA testing if publicly available?
   a. Yes
   b. No
5) Patient F's ctDNA status was tested following surgery. If the patient's ctDNA status was **negative** would you prescribe adjuvant chemotherapy?
   a. Yes
   b. No
6) Please select the chemotherapy regimen you would recommend to Patient F if the ctDNA status was **negative**:
   a. 5-FU
   b. Capecitabine
   c. FOLFOX
   d. CAPOX
7) Please select the duration of chemotherapy you would recommend:
   a. 3 months
   b. 6months
8) Patient F's ctDNA status was tested following surgery. If the patient's ctDNA status was **positive** would you prescribe adjuvant chemotherapy?
   a. Yes
   b. No
9) Please select the chemotherapy regimen you would recommend to Patient F if the ctDNA status was **positive**:
   a. 5-FU
   b. Capecitabine
   c. FOLFOX
   d. CAPOX
10) Please select the duration of chemotherapy you would recommend:
   a. 3 months
   b. 6months

Vignette 7:
Patient G is ≥ **70 years old** with a recently resected stage II colon cancer. Clinicopathological risk for recurrence: **Intermediate risk**

1) Would you prescribe adjuvant chemotherapy to Patient G?
    a. Yes – *please note question 2 and 3 only becomes visible if "Yes" is selected*
    b. No
2) Please select the chemotherapy regimen you would recommend for Patient G:
    a. 5-FU
    b. Capecitabine
    c. FOLFOX
    d. CAPOX
3) Please select the duration of chemotherapy you would recommend:
    a. 3 months
    b. 6 months
4) Following surgery, for Patient G would you order ctDNA testing if publicly available?
    a. Yes
    b. No
5) Patient G's ctDNA status was tested following surgery. If the patient's ctDNA status was **<u>negative</u>** would you prescribe adjuvant chemotherapy?
    a. Yes
    b. No
6) Please select the chemotherapy regimen you would recommend to Patient G if the ctDNA status was **<u>negative</u>**:
    a. 5-FU
    b. Capecitabine
    c. FOLFOX
    d. CAPOX
7) Please select the duration of chemotherapy you would recommend:
    a. 3 months
    b. 6months
8) Patient G's ctDNA status was tested following surgery. If the patient's ctDNA status was **<u>positive</u>** would you prescribe adjuvant chemotherapy?
    a. Yes
    b. No
9) Please select the chemotherapy regimen you would recommend to Patient G if the ctDNA status was **<u>positive</u>**:
    a. 5-FU
    b. Capecitabine
    c. FOLFOX
    d. CAPOX
10) Please select the duration of chemotherapy you would recommend:
    a. 3 months
    b. 6months

Vignette 8:
Patient H is **between 51 to 69 years old** with a recently resected stage II colon cancer. Clinicopathological risk for recurrence: **Low risk**

1) Would you prescribe adjuvant chemotherapy to Patient H?
    a. Yes – *please note question 2 and 3 only becomes visible if "Yes" is selected*

b. No
2) Please select the chemotherapy regimen you would recommend for Patient H:
    a. 5-FU
    b. Capecitabine
    c. FOLFOX
    d. CAPOX
3) Please select the duration of chemotherapy you would recommend:
    a. 3 months
    b. 6 months
4) Following surgery, for Patient H would you order ctDNA testing if publicly available?
    a. Yes
    b. No
5) Patient H's ctDNA status was tested following surgery. If the patient's ctDNA status was **<u>negative</u>** would you prescribe adjuvant chemotherapy?
    a. Yes
    b. No
6) Please select the chemotherapy regimen you would recommend to Patient H if the ctDNA status was **<u>negative</u>**:
    a. 5-FU
    b. Capecitabine
    c. FOLFOX
    d. CAPOX
7) Please select the duration of chemotherapy you would recommend:
    a. 3 months
    b. 6months
8) Patient H's ctDNA status was tested following surgery. If the patient's ctDNA status was **<u>positive</u>** would you prescribe adjuvant chemotherapy?
    a. Yes
    b. No
9) Please select the chemotherapy regimen you would recommend to Patient H if the ctDNA status was **<u>positive</u>**:
    a. 5-FU
    b. Capecitabine
    c. FOLFOX
    d. CAPOX
10) Please select the duration of chemotherapy you would recommend:
    a. 3 months
    b. 6months

Vignette 9:
Patient I is $\leq$ **50 years old** with a recently resected stage II colon cancer. Clinicopathological risk for recurrence: **Intermediate risk**

1) Would you prescribe adjuvant chemotherapy to Patient I?
    a. Yes – *please note question 2 and 3 only becomes visible if "Yes" is selected*
    b. No
2) Please select the chemotherapy regimen you would recommend for Patient I:
    a. 5-FU
    b. Capecitabine
    c. FOLFOX

d. CAPOX

3) Please select the duration of chemotherapy you would recommend:
   a. 3 months
   b. 6 months
4) Following surgery, for Patient I would you order ctDNA testing if publicly available?
   a. Yes
   b. No
5) Patient I's ctDNA status was tested following surgery. If the patient's ctDNA status was **negative** would you prescribe adjuvant chemotherapy?
   a. Yes
   b. No
6) Please select the chemotherapy regimen you would recommend to Patient I if the ctDNA status was **negative**:
   a. 5-FU
   b. Capecitabine
   c. FOLFOX
   d. CAPOX
7) Please select the duration of chemotherapy you would recommend:
   a. 3 months
   b. 6months
8) Patient I's ctDNA status was tested following surgery. If the patient's ctDNA status was **positive** would you prescribe adjuvant chemotherapy?
   a. Yes
   b. No
9) Please select the chemotherapy regimen you would recommend to Patient I if the ctDNA status was **positive**:
   a. 5-FU
   b. Capecitabine
   c. FOLFOX
   d. CAPOX
10) Please select the duration of chemotherapy you would recommend:
   a. 3 months
   b. 6months

9.5 Part 4: End of survey

We thank you for your time spent taking this survey. Your responses has been recorded.

References

1. Eysenbach G. Improving the Quality of Web Surveys: The Checklist for Reporting Results of Internet E-Surveys (CHERRIES). J Med Internet Res. 2004;6(3):e34.
